# Isolation and cultivation of a novel sulfate-reducing magnetotactic bacterium belonging to the genus *Desulfovibrio*

**DOI:** 10.1371/journal.pone.0248313

**Published:** 2021-03-11

**Authors:** Hirokazu Shimoshige, Hideki Kobayashi, Shigeru Shimamura, Toru Mizuki, Akira Inoue, Toru Maekawa

**Affiliations:** 1 Bio-Nano Electronics Research Centre, Toyo University, Kawagoe, Saitama, Japan; 2 Japan Agency for Marine-Earth Science and Technology, Yokosuka, Kanagawa, Japan; 3 Graduate School of Interdisciplinary New Science, Toyo University, Kawagoe, Saitama, Japan; Universidade Nova de Lisboa, PORTUGAL

## Abstract

Magnetotactic bacteria (MTB) synthesize magnetosomes composed of membrane-enveloped magnetite (Fe_3_O_4_) and/or greigite (Fe_3_S_4_) nanoparticles in the cells. It is known that the magnetotactic *Deltaproteobacteria* are ubiquitous and inhabit worldwide in the sediments of freshwater and marine environments. Mostly known MTB belonging to the *Deltaproteobacteria* are dissimilatory sulfate-reducing bacteria that biomineralize bullet-shaped magnetite nanoparticles, but only a few axenic cultures have been obtained so far. Here, we report the isolation, cultivation and characterization of a dissimilatory sulfate-reducing magnetotactic bacterium, which we designate “strain FSS-1”. We found that the strain FSS-1 is a strict anaerobe and uses casamino acids as electron donors and sulfate as an electron acceptor to reduce sulfate to hydrogen sulfide. The strain FSS-1 produced bullet-shaped magnetite nanoparticles in the cells and responded to external magnetic fields. On the basis of 16S rRNA gene sequence analysis, the strain FSS-1 is a member of the genus *Desulfovibrio*, showing a 96.7% sequence similarity to *Desulfovibrio putealis* strain B7-43^T^. Futhermore, the magnetosome gene cluster of strain FSS-1 was different from that of *Desulfovibrio magneticus* strain RS-1. Thus, the strain FSS-1 is considered to be a novel sulfate-reducing magnetotactic bacterium belonging to the genus *Desulfovibrio*.

## Introduction

Magnetotactic bacteria (MTB) are Gram-negative prokaryotes that synthesize intracellular magnetic nanoparticles named magnetosomes. Magnetosomes are membrane-bounded crystals, which are composed of magnetite (Fe_3_O_4_) and/or greigite (Fe_3_S_4_) and characterized by the narrow size distribution in each cell ranging from 30 to 280 nm, distinct species-specific crystal morphology and chemical purity, form aligned structures, arranging a single or multiple linear chains within the cells [1–9]. Mostly known MTB are affiliated with the *Alphaproteobacteria*, *Gammaproteobacteria*, *Deltaproteobacteria* and *Etaproteobacteria* classes of the *Proteobacteria* phylum, and with the *Nitrospirae* and “*Candidatus* Omnitrophica” phyla [1, 9–11]. The existences of MTB belonging to the *Zetaproteobacteria* and “*Candidatus* Lamdaproteobacteria” classes have also recently been clarified by metagenomic analysis [12]. MTB belonging to the *Deltaproteobacteria* class are known to synthesize bullet-shaped magnetite and/or pleomorphic greigite within the same cells [3, 5, 13]. Magnetotactic *Deltaproteobacteria* have been widely found in sediments in the sea, river estuaries, salt ponds, lagoons, and alkaline and freshwater environments [14–20]. It was reported that rich diversity of magnetotactic *Deltaproteobacteria* inhabit in a freshwater environment [21]. Magnetotactic *Deltaproteobacteria* are located close to the root of the phylogenetic tree of the *Proteobacteria*, which synthesize only bullet-shaped magnetite [5]. The magnetotactic *Nitrospirae* and *Omnitrophica*, which are known to produce only bullet-shaped magnetite, represent the deep-branching MTB groups [22–25]. It has recently been demonstrated that the crystal habit and growth pattern of the bullet-shaped magnetite formed by magnetotactic *Nitrospirae* were quite different from those formed by the magnetotactic *Deltaproteobacteria* [8, 26]. The bullet-shaped magnetite nanoparticles have attracted a lot of attention from microbiological and geological researchers since the magnetotactic *Deltaproteobacteria* generally synthesize a large number of magnetite nanoparticles in each cell, which contributes to sedimentary magnetization [27, 28].

Magnetotactic *Deltaproteobacteria* are present in two orders; the *Desulfovibrionales* and the *Desulfobacterales* [5, 29, 30]. *Desulfovibrio magneticus* strain RS-1^T^ associated with the order *Desulfovibrionales*, which was isolated for the first time from a waterway near Kameno River, Wakayama, Japan, is a dissimilatory sulfate-reducing bacterium (SRB) that synthesizes bullet-shaped magnetite nanoparticles in the cell [30]. Strain FH-1 and strain ZBP-1 belonging to the genus *Desulfovibrio* are also dissimilatory sulfate-reducing bacteria that produce bullet-shaped magnetite nanoparticles [31]. Strain ML-1, strain AV-1 and strain ZZ-1 of *Desulfonatronum thiodismutans* belonging to the order *Desulfovibrionales* are obligately alkaliphilic and sulfate-reducing MTB, the optimal pH for the growth of which is 9.0–9.5 [15]. There are three magnetotactic multicellular prokaryotes (MMPs) associated with the order *Desulfobacterales*, which are tentatively named “*Candidatus* Magnetoglobus multicellularis”, “*Ca*. Magnetomorum litorale”, and “*Ca*. Magnetananas tsingtaoensis” [29, 32, 33]. It is believed that those MMPs are most likely sulfate-reducing bacteria, which synthesize pleomorphic greigite and/or bullet-shaped magnetite [16, 32, 33]. *Desulfamplus magnetovallimortis* strain BW-1^T^ belonging to the order *Desulfobacterales* was isolated from a brackish spring in Death Valley National Park, California, USA, and cultivated in axenic culture [5, 34]. *D*. *magnetovallimortis* is a sulfate-reducing bacterium that can produce both bullet-shaped magnetite and pleomorphic greigite within the same cell [5]. Strain WYHR-1 is a magnetotactic deltaproteobacterium that produces bullet-shaped magnetite nanoparticles, the crystal morphology of which is quite different from that of the magnetite nanoparticles produced by *Deltaproteobacteria* and magnetotactic *Nitrospirae* [20]. It was recently reported that ectosymbiotic magnetotactic *Deltaproteobacteria* observed in marine anoxic sediments are likely to be sulfate-reducing bacteria [35].

SRB are generally known to be strictly anaerobic and use a variety of organic compounds or molecular hydrogen (H_2_) to obtain energy for growth, oxidizing organic compounds or H_2_ and reducing sulfate to hydrogen sulfide (H_2_S) [36]. In other words, they use organic compounds or H_2_ as electron donors and sulfate as an electron acceptor [36]. Cultured magnetotactic *Deltaproteobacteria* also use organic acids, alcohol or hydrogen as electron donors in the presence of sulfate as an electron acceptor. *D*. *magneticus* uses lactate, pyruvate, malate, ethanol and glycerol as electron donors and carbon sources, and sulfate as an electron acceptor [30]. All of the strains of *D*. *thiodismutans* use formate and hydrogen as electron donors, while using sulfate as an electron acceptor [15]. *D*. *magnetovallimortis* uses lactate, pyruvate, fumarate, succinate and malate as electron donors, and sulfate as an electron acceptor [34]. It has been shown that several strains of SRB can use amino acids as electron donors, and sulfate as an electron acceptor [37–40]. Some SRB inhabiting in freshwater sediments are likely to use amino acids as electron donors for sulfate reduction [41]. It was also reported that SRB actively use amino acids, noting that sulfate reduction was more stimulated by the addition of casamino acids to marine sediments than lactate [42]. Therefore, there is a possibility that a large number of sulfate-reducing MTB, which use amino acids as electron donors, may be widely distributed in sediments of natural aquatic environments.

In this study, we isolated and cultivated a novel sulfate-reducing magnetotactic bacterium from freshwater and sediments of a pond, using casamino acids as electron donors. We successfully isolated “strain FSS-1”, which is a dissimilatory sulfate-reducing bacterium, and found that bullet-shaped magnetite nanoparticles were synthesized in each cell, using several amino acids as electron donors in the presence of sulfate as an electron acceptor. The phylogenetic analysis based on 16S rRNA gene sequences and the genome sequence analysis of the magnetosome gene cluster showed that the strain FSS-1 is considered to be a novel sulfate-reducing magnetotactic bacterium belonging to the genus *Desulfovibrio*.

## Materials and methods

### Screening and cultivation of strain FSS-1

Sediments together with freshwater, the ratio of which was 1:2, were collected from Suwa Pond in Hidaka, Saitama, Japan (35.892°N, 139.368°E) in June 2017, and transferred to 2-liter plastic bottles. Note that no permits were required for the collection of the samples from Hidaka City. MTB were enriched by neodymium-boron magnets (ϕ10 × 10 mm) of 0.48 T, attaching them to the outer surface of the bottles at 1 cm above the sediment-water interface for 60 min, and then the cells accumulated by the magnets were collected with a Pasteur pipette and transferred to test tubes. The MTB cells were then magnetically concentrated by an MTB trap device for 120 min [43]. Modifying the medium previously used for the cultivation of *Desulfovibrio* [31], we developed a new one named “magnetotactic *Desulfovibrio* medium”, abbreviated to “MD medium”, which was composed of 0.5 mL/liter of modified Wolfe’s mineral elixir [44, 45], 0.5 mg/liter of resazurin, 0.25 g/liter of NH_4_Cl, 0.1 g/liter of MgSO_4_•7H_2_O, 0.1 g/liter of casamino acids (Becton, Dickinson and Company), and 0.02 g/liter of yeast extract (Becton, Dickinson and Company), and the pH of the medium was adjusted to 7.0. The MTB enriched by the MTB trap device were inoculated in screw-capped glass culture tubes as follows; (i) The screw-capped glass culture tubes were filled up to approximately 66% of their volume with the MD medium, which was then bubbled with 100% O_2_ free-N_2_ gas for 5 min and autoclaved; (ii) After having autoclaved the medium, 0.5 ml/liter of a sterile anaerobic stock of vitamin solution [46], 5.6 ml/liter of a sterile anaerobic stock of 0.25 mM KHPO_4_ buffer (pH 7.0), 5.0 ml/liter of a sterile anaerobic stock of 10 mM ferric quinate, and 0.4 g/liter of freshly made neutralized and filter sterilized cysteine•HCl•H_2_O were added to the medium; (iii) Strain FSS-1 was incubated in the MD medium at 28°C in dim light. An axenic culture of cells was then obtained by the MTB trap device, followed by dilution to extinction. We also carried out a test for the growth of strain FSS-1 under microaerobic conditions following the procedure employed by Lefèvre et al. [31].

### Optical and Transmission Electron Microscopic (TEM) observation of strain FSS-1

Response of strain FSS-1 cells grown in the MD medium to an external magnetic field was checked by an optical microscope (DM5000B, LEICA) using a ferrite magnet (50 mm × 14 mm × 10 mm) (Niroku Seisakusho). The cells at a stationary phase were placed on a TEM grid (200 mesh Cu Formvar/carbon-coated grid, JEOL) and air-dried at room temperature. The grid was rinsed twice with sterile distilled water and then the cells were observed by a TEM (JEM-2100, JEOL) with an accelerating voltage of 160 kV.

### Sequence and phylogenetic analysis of strain FSS-1

DNA was extracted from strain FSS-1 using DNeasy (QIAGEN) and the 16S rRNA gene was amplified using the universal bacterial primers 27F (5′-AGAGTTTGATCCTGGCTCAG-3′) and 1492R (5′-GGTTACCTTGTTACGACTT-3′) [47]. The PCR reaction was carried out as follows; the template DNA was initially denatured at 95°C for 2 min, followed by 25 cycles of the temperature control; i.e., 95°C for 20 s, 50°C for 30 s and 72°C for 90 s, and a final extension step at 72°C for 5 min. The PCR product was purified using a QIAquick PCR Purification Kit (QIAGEN) and cloned into the pCR2.1 T vector using a TA Cloning Kit (Invitrogen) and chemically competent cells of *Escherichia coli* DH5α (TaKaRa). The transformed cells were incubated overnight at 37°C on LB agar plates with 100 μg/ml ampicillin. The clone was sequenced using an ABI3130xl genetic analyzer with Big Dye ver3.1 following the manufacturer’s instruction (Applied Biosystems). The obtained sequences were assembled and analyzed with Sequencher ver 4.10.1 (Gene Codes). The 16S rRNA genes sequences of strain FSS-1 obtained in this study have been deposited in the DDBJ/EMBL/GenBank database under the following accession number: LC311577. The 16S rRNA gene sequences of related strains retrieved from the DNA Data Bank of Japan were aligned using the CLUSTAL X 2.0.12 multiple alignment accessory application [48–51]. A phylogenetic tree was reconstructed using the neighbor-joining (NJ) method and evaluated by bootstrap sampling [52, 53]. The NJ tree was drawn using NJplot 2.1.

### Growth of strain FSS-1 in the presence of casamino acids

The growth of strain FSS-1 was investigated in the MD medium containing 0.025, 0.05, 0.1, 0.2 and 0.4 g/liter of casamino acids in the absence of sodium sulfate, and 0.1, 0.2, 0.4 and 0.8 g/liter of sodium sulfate in the presence of 0.1 g/liter casamino acids under anaerobic conditions at 28°C in dim light. The growth curve of strain FSS-1 in the MD medium containing 0.1 g/liter of casamino acids under anaerobic conditions was obtained using a bacterial counting chamber (Erma).

### Energy-Dispersive X-Ray Spectrometric (EDS) analysis and High-Resolution TEM (HRTEM) observation of magnetic nanoparticles in the cells

Scanning TEM (STEM)-EDS and HRTEM observation was performed using a TEM (JEM-2200FS equipped with JED-2300T EDS system, JEOL) operated at 200 kV. The number of magnetic nanoparticles in each cell was counted targeting at 50 individual cells. The size of magnetic nanoparticles was measured based on 466 magnetic nanoparticles from several TEM micrographs using Digital Micrograph software (Gatan). We also obtained fast Fourier transform (FFT) patterns using Digital Micrograph software (Gatan).

### Genome sequencing and comparative analysis of the Magnetosome Gene Cluster (MGC) of strain FSS-1

Genome sequencing analysis was conducted at the Techno Suruga Co. Ltd (Shizuoka, Japan). The genomic DNA was extracted from strain FSS-1 and prepared the sequencing library (Nextera DNA Flex Library Kit, Illumina and Nextera DNA CD Indexes, Illumina) for paired-end 2×151 bp sequencing using an iSeq 100 (Illumina). Illumina reads were trimmed to remove the adapter sequences and low-quality bases, and assembled using IDBA-UD ver 1.1.2 [54]. The quality and accuracy of the acquired genomic DNA sequence were assessed using FASTX-Toolkit ver 0.0.14 [55]. The magnetosome genes were checked and verified manually using blastx of the National Center for Biotechnology Information Basic Local Alignment Search Tool (NCBI BLAST). The *mam* genes, *mad* genes and other predicted genes of strain FSS-1 were compared with *Desulfovibrio magneticus* strain RS-1^T^ (AP010904) [56, 57]. The sequence of the MGC of strain FSS-1 has been submitted to the DDBJ/EMBL/GenBank database under the following accession number: BLTE01000001. Amino acid sequences of Mam and Mad proteins identified from strain FSS-1 were used for *E*-value, coverage and identity analyses using the blastx of the NCBI compared with those of all magnetotactic bacteria.

## Results

### Isolation of strain FSS-1

**Fig 1:**
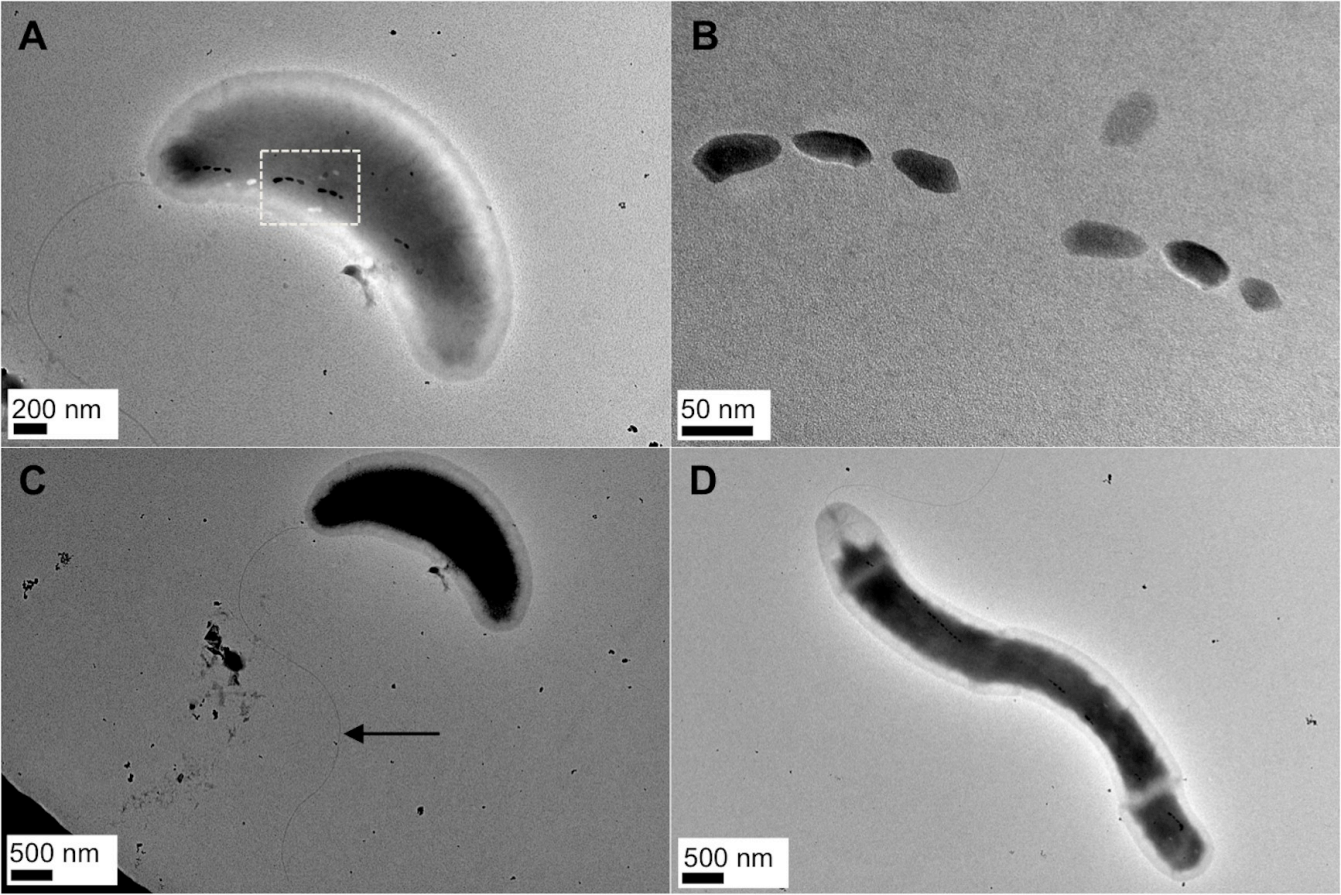
Morphological features of strain FSS-1. (A) TEM image of a vibroid cell of strain FSS-1 incubated in the MD medium under anaerobic conditions. (B) TEM image of a magnetosome synthesized in the cell corresponding to the dashed-line box indicated in panel (A). (C) TEM image of the same cell as shown in panel (A). A single polar flagellum is indicated by a black arrow. (D) TEM image of a spiral cell of strain FSS-1.

MTB were magnetically collected from freshwater sediments of Suwa Pond in Hidaka, Saitama, Japan, and inoculated into the MD medium under anaerobic conditions as mentioned. After 6 day incubation at 28°C, the growth of MTB collected from the freshwater sediments was confirmed by detecting the production of ferrous sulfide (FeS). Optical microscopic observation of the cells in culture tubes using a magnet showed that they were magnetotactic, and the morphology was either vibroid or spiral. To obtain an axenic culture, the MTB in the culture were enriched by the modified MTB trap device and was diluted to extinction. In order to confirm the axenic culture, we observed the isolate, which we designate “strain FSS-1”, by an optical microscope and TEM. We confirmed that bullet-shaped magnetosomes were formed in the cells (see Fig 1A and 1B). TEM images show that the morphology of the cells was either vibroid or spiral with a width and length of 0.8 ± 0.1 μm and 2.9 ± 0.9 μm, where the number of sampled cells was 50, and each cell possessed a single polar flagellum (Fig 1A, 1C and 1D). We confirmed that strain FSS-1 was a strict anaerobe (S1 Fig).

**Fig 2:**
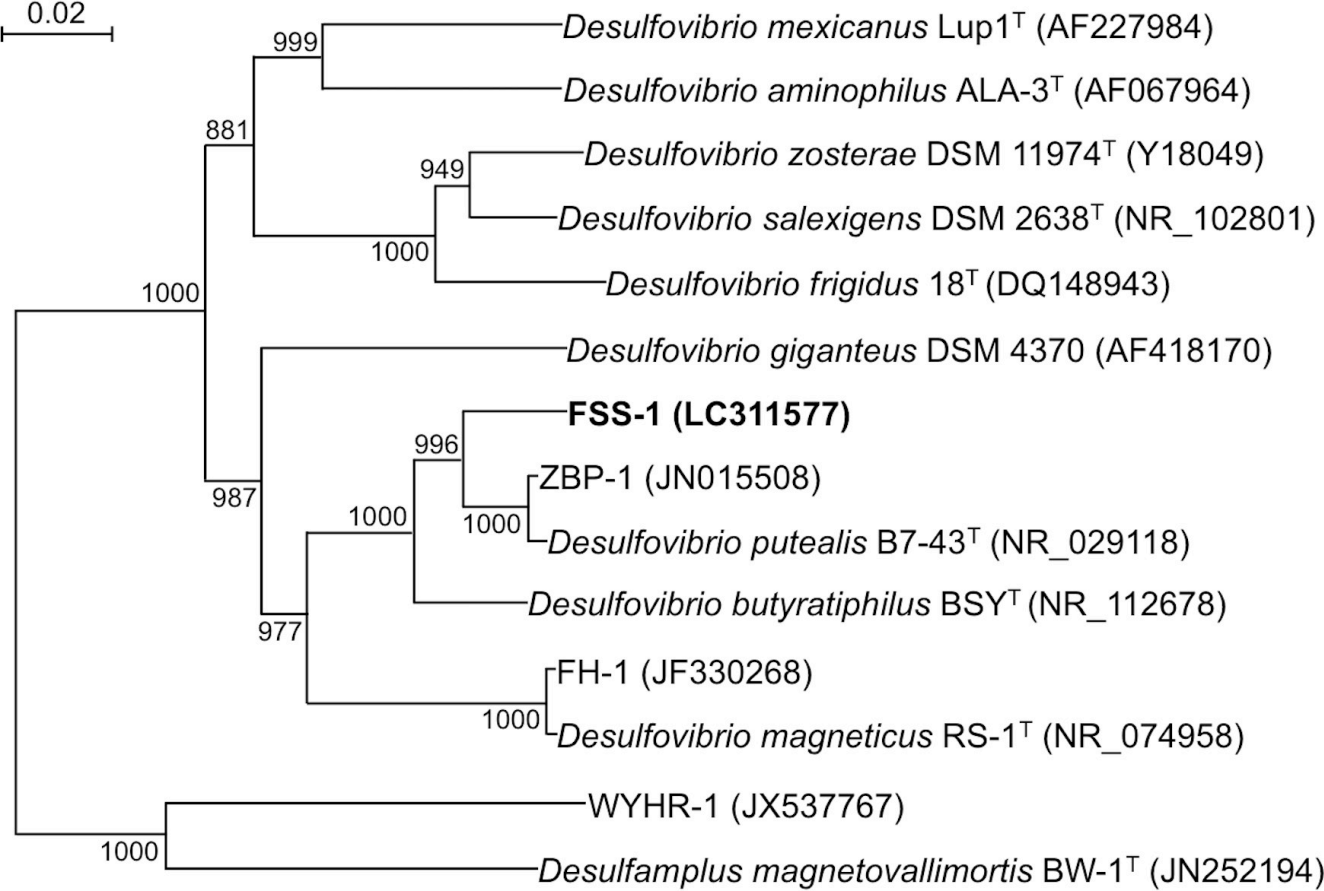
Phylogenetic tree based on the 16S rRNA gene sequences of strain FSS-1 and some other related *Desulfovibrio*. The tree was constructed by the neighbor-joining method and rooted using *Escherichia coli* as an outgroup. Bootstrap values per 1,000 replicates are indicated. The GenBank accession numbers are shown in the parentheses. Bar, 0.01 changes per nucleotide position.

The neighbor-joining tree shows that the strain FSS-1 is a member of the family *Desulfovibrionaceae* of *Deltaproteobacteria* and is most closely related to *Desulfovibrio putealis* strain B7-43^T^ (Fig 2). According to the similarity search performed using blastn of the NCBI BLAST, the 16S rRNA gene sequence of the strain FSS-1 is also most closely related to *D*. *putealis* strain B7-43^T^, the mutual genes having shown 96.7% similarity. The sequence of the strain FSS-1 showed 96.6%, 91.6% and 91.4% similarity, respectively, with *Desulfovibrio sp*. strain ZBP-1, *Desulfovibrio sp*. strain FH-1 and *D*. *magneticus* strain RS-1^T^. Based on these 16S rRNA gene sequence similarities, the strain FSS-1 is considered to be a novel species of the genus *Desulfovibrio*.

### Growth of strain FSS-1 in the presence of casamino acids

**Fig 3:**
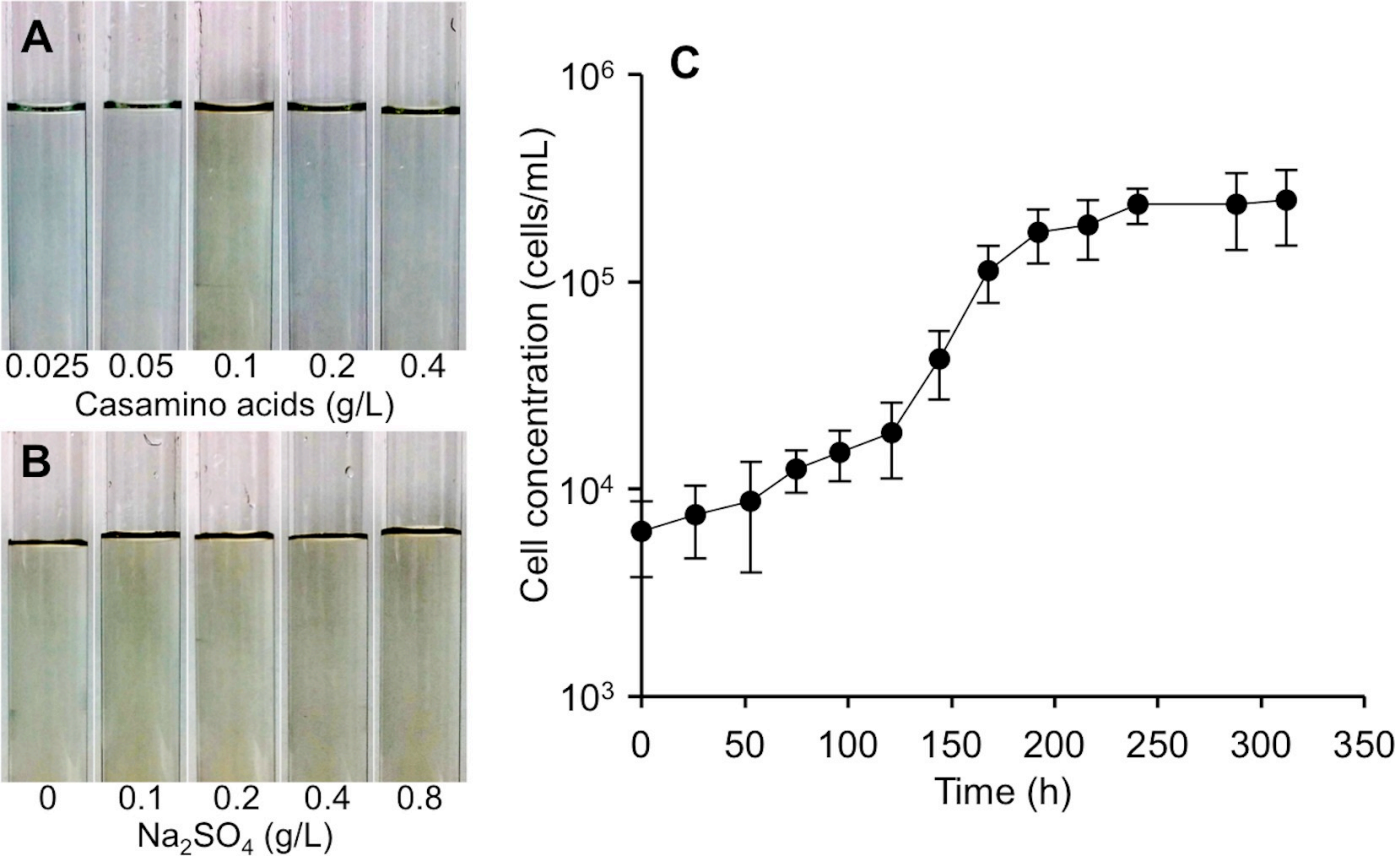
Growth of strain FSS-1 in the MD medium containing casamino acids and sodium sulfate. (A) MD medium containing 0.025, 0.05, 0.1, 0.2 and 0.4 g/liter of casamino acids after 13 day incubation. Cells grew only when 0.1 g/liter of casamino acids was present in the medium as indicated by the production of ferrous sulfide. (B) MD containing 0.1, 0.2, 0.4 and 0.8 g/liter of sodium sulfate in the presence of 0.1 g/liter casamino acids after 13 day incubation. Cells grew irrespective of the difference in the concentration of sodium sulfate in the medium. (C) Growth curve of strain FSS-1 in the liquid medium containing 0.1 g/liter of casamino acids in the absence of sodium sulfate. The average values were calculated from three independent experiments. The error bars represent the standard deviations.

To investigate the effect of the concentration of casamino acids and sulfate on the growth of strain FSS-1, the cells were grown in the MD medium containing different concentrations of casamino acids and sodium sulfate. After 13 day incubation at 28°C, the color of the liquid medium turned to yellowish brown only in the case of 0.1 g/liter of casamino acids, noting that the color change was caused by the production of ferrous sulfide during the growth (Fig 3A), whereas the color changed to yellowish brown irrespective of the difference in the concentration of sodium sulfate (Fig 3B). It is therefore supposed that the optimal concentration of casamino acids for the growth of FSS-1 cells is 0.1 g/liter. The growth curve of strain FSS-1 in the MD medium, which contained 0.1 g/liter of casamino acids and no sodium sulfate, is shown in Fig 3C. The doubling time during the exponential growth phase in the presence of 0.1 g/liter of casamino acids was approximately 18 h and the final cell concentration was 2.5 × 10^5^ cells/mL.

### Formation of bullet-shaped magnetic nanoparticles in strain FSS-1

**Fig 4:**
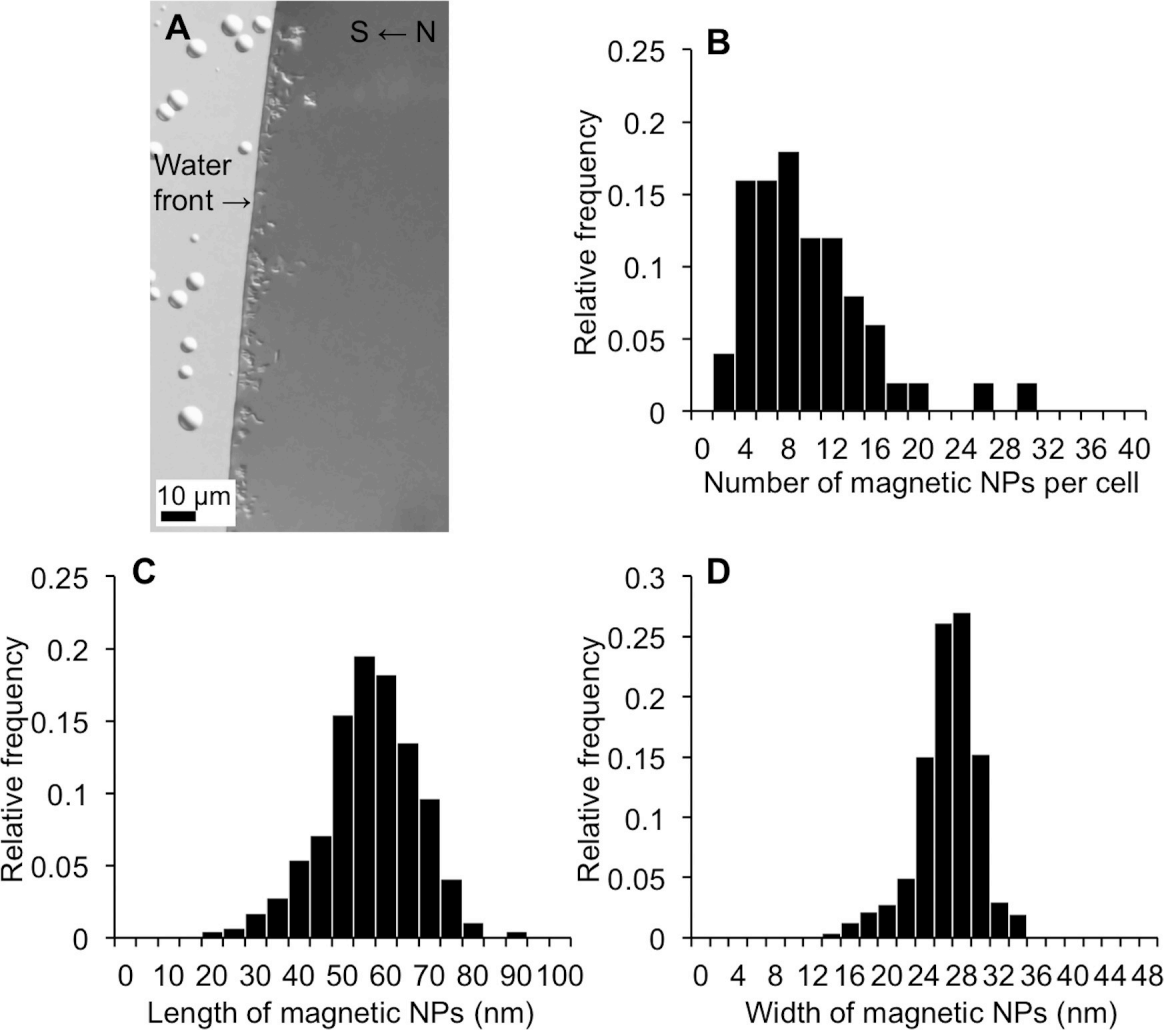
Response of strain FSS-1 cells to an external magnetic field, the number of magnetic nanoparticles in each cell and the size distribution of magnetic nanoparticles. (A) Differential interface contrast (DIC) optical microscopic image of strain FSS-1 cells at the edge of a ‘hanging drop’ in an external magnetic field. (B) Distribution of the number of magnetic nanoparticles in each cell. (C) Distribution of the length of magnetic nanoparticles. (D) Distribution of the width of magnetic nanoparticles.

Strain FSS-1 cells grown in the MD medium containing 0.1 g/liter of casamino acids showed north-seeking magnetotactic behavior judging by the fact that almost all of the cells preferentially swam parallel to the external magnetic field lines toward the south pole of the ferrite magnet (Fig 4A). The number of magnetic nanoparticles per cell grown in the presence of 0.1 g/liter of casamino acids for 13 days was 9.4 ± 5.7 (mean ± SD, range 2–28) (Fig 4B). The length and width of magnetic nanoparticles synthesized in the presence of 0.1 g/liter of casamino acids were, respectively, 53.9 ± 11.0 nm (mean ± SD, range 19.3–88.7) and 25.5 ± 3.3 nm (mean ± SD, range 12.7–34.0) (Fig 4C and 4D). We observed electron-dense precipitates from a culture of strain FSS-1 grown in the MD medium containing 0.1 g/liter of casamino acids (S2 Fig). Phosphorus (P), oxygen (O), iron (Fe) and sulfur (S) were mainly detected from the electron-dense precipitates by EDS analysis (S2 Fig). It is supposed that the precipitates, which were not associated with magnetosomes, were composed of ferric phosphate and ferrous sulfide, noting that ferric phosphate was formed by iron and phosphate contained in the liquid medium, whereas the ferrous sulfide was produced via sulfate reduction during the growth. To examine the effect of an individual amino acid included in casamino acids on the growth of strain FSS-1 and magnetosome formation, the cells were grown in the MD medium containing a single amino acid as an electron donor in the presence of sulfate (S1 Table). We found that strain FSS-1 was successfully grown in the presence of asparaginic acid, glycine, tryptophan and valine as electron donors and sulfate as an electron acceptor and synthesized bullet-shaped magnetic nanoparticles (S1 Table).

### EDS analysis and HRTEM observation of magnetic nanoparticles in the cell

**Fig 5:**
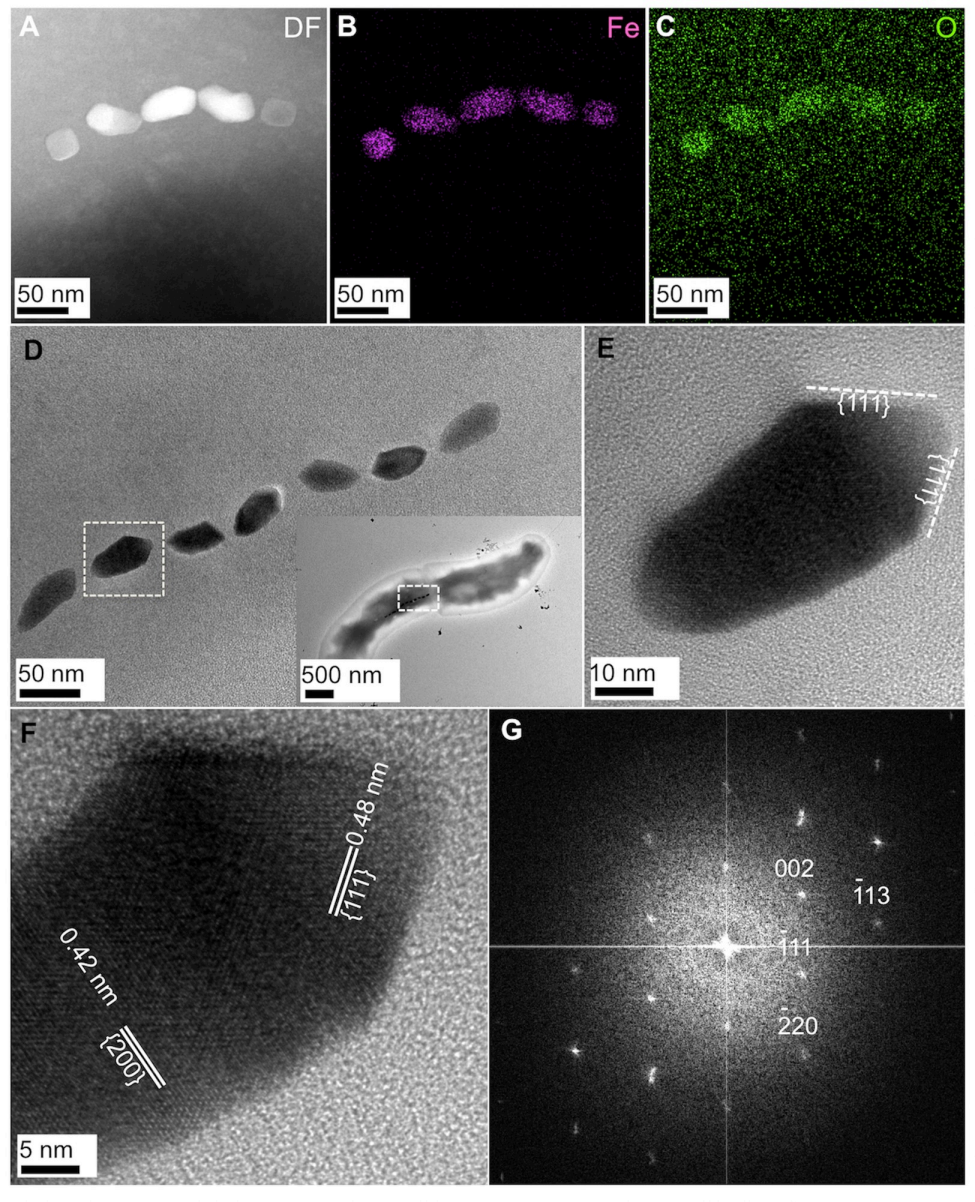
STEM-EDS analysis, and TEM and HRTEM images of magnetic nanoparticles in each cell. (A) STEM image of a magnetosome. (B), (C) STEM-EDS elemental maps corresponding to iron (Fe) and oxygen (O). (D) TEM image of a magnetosome in a cell corresponding to the dashed-line box indicated in the inset. (E) HRTEM image of a magnetic nanoparticle indicated by the dashed-line box in panel (D). (F) HRTEM image of the same nanoparticle as shown in panel (E). (G) Fast Fourier transform (FFT) pattern of the same nanoparticle as shown in panel (F).

We performed EDS analysis of the magnetic nanoparticles in the cells (Fig 5A–5C and S3 Fig). Spot EDS spectra and STEM-EDS elemental maps show that iron (Fe) and oxygen (O) were mainly detected from the magnetic nanoparticles (Fig 5A–5C and S3 Fig). Thus, it is supposed that the magnetic nanoparticles are either magnetite or maghemite.

HRTEM observation of the magnetic nanoparticles in a cell reveals that the magnetite was synthesized, identified from both fast Fourier transform (FFT) and lattice spacing analyses (Fig 5D–5G). The HRTEM images show that the elongation direction of the bullet-shaped magnetite nanoparticles in the cells was not parallel to the <111> direction (Fig 5D and 5E). The *d* spacing values of the magnetic nanoparticles were 0.48 and 0.42 nm, which were in accordance with {111} and {200} of face-centered cubic magnetite (Fig 5F and 5G).

### Magnetosome Gene Cluster (MGC) of strain FSS-1

**Fig 6:**
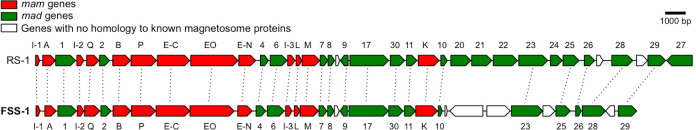
Comparison of Magnetosome Gene Clusters (MGCs) between strain FSS-1 and *Desulfovibrio magneticus* strain RS-1^T^. 13 *mam* genes, 16 *mad* genes and 6 non-homologous genes are present in the MGC of strain FSS-1. Dashed-lines between two MGCs show homologous genes.

Genome sequencing analysis of MGC of strain FSS-1 revealed a genomic region of 29.486 kb that contained 13 *mam* genes (*mamI-1*, *mamA*, *mamI-2*, *mamQ*, *mamB*, *mamP-like*, *mamE-Cter*, *mamEO*, *mamE-Nter*, *mamI-3*, *mamL*, *mamM*, *mamK*) and 16 *mad* genes (*mad1*, *mad2*, *mad4*, *mad6*, *mad7*, *mad8*, *mad9*, *mad17*, *mad30*, *mad11*, *mad10*, *mad23*, *mad25*, *mad26*, *mad28*, *mad29*) (Fig 6, Table 1). The MGC started with the *mamI-1* gene and ended with the *mad29* gene. The MGC also contained 6 genes with no homology to known magnetosome proteins, 3 with high amino acid sequence similarities to 3 hypothetical proteins in the MGC of *D*. *magneticus* strain RS-1, 1 (one) with a high amino acid sequence similarity to a hypothetical protein in the genome of *D*. *magneticus* strain RS-1 and 2 without any similarities to known proteins. Transposase genes or insertion elements were not found within 10 kb upstream of *mamI-1* gene and 10 kb downstream of *mad29* gene. Comparative analysis of the MGC indicated that the *mad* genes and the above non-homologous genes of strain FSS-1 differed from those of *Desulfovibrio magneticus* strain RS-1^T^ (Fig 6). The *mam* 20, *mad* 21, *mam* 22, *mad* 24 and *mad* 27 genes of *D*. *magneticus* strain RS-1^T^ were not found in the MGC of strain FSS-1. Putative *mad27* gene (1527 bp) was found in 3061 bp upstream of the *mamI-1* gene. The putative *mad27* gene showed a very close similarity to the *mad27* gene of *Desulfovibrio* sp. strain FH-1 (AGG16231.1), which was resulted from the high amino acid sequence similarity analysis using blastx of the NCBI (identity 49%, coverage 78%, *E*-value 2e-97). The G+C content of the 13 *mam* genes of strain FSS-1 was 64.2%.

**Table 1:** Comparison of known Mam and Mad proteins identified from the genome of strain FSS-1 with those of other magnetotactic bacteria.

***Ca*. Magnetomorum sp. HK-1**	**Identity (%)**	60	46	47	38	56	44	44	−	50	45	49	35	−	44	69	−	52	−	−	49	−	−	−	33	−	−	−	−	−
	**Coverage (%)**	91	95	99	99	87	98	78	−	97	56	61	87	−	76	92	−	93	−	−	93	−	−	−	87	−	−	−	−	−
	***E*-value**	9e-15	6e-55	2e-19	4e-50	5e-100	4e-56	1e-99	−	7e-72	1e-07	4e-04	7e-39	−	2e-36	2e-60	−	2e-76	−	−	3e-33	−	−	−	2e-15	−	−	−	−	−
***Desulfonatronum* sp. ML-1**	**Identity (%)**	68	−	57	−	71	48	50	52	−	38	59	51	71	48	−	44	54	43	52	59	−	−	31	52	44	47	−	−	−
	**Coverage (%)**	75	−	99	−	97	98	76	30	−	93	98	87	99	73	−	84	98	93	70	96	−	−	89	91	78	52	−	−	−
	***E*-value**	5e-11	−	8e-28	−	1e-122	2e-109	2e-137	1e-67	−	6e-06	1e-06	5e-75	0.0	1e-32	−	3e-22	8e-93	2e-09	2e-05	3e-51	−	−	8e-07	8e-35	5e-82	4e-23	−	−	−
***Desulfovibrio* sp. FH-1**	**Identity (%)**	−	68	−	48	70	59	50	59	−	45	61	49	−	55	−	46	58	38	55	65	59	60	−	−	−	45	46	69	59
	**Coverage (%)**	−	98	−	99	87	93	89	30	−	92	50	85	−	74	−	44	98	91	70	94	72	88	−	−	−	85	69	98	96
	***E*-value**	−	4e-95	−	1e-69	2e-131	6e-139	4e-154	4e-80	−	1e-11	0.004	2e-69	−	2e-31	−	7e-05	2e-104	9e-08	1e-16	3e-52	8e-153	1e-72	−	−	−	3e-29	6e-10	0.0	8e-107
***Desulfovibrio magneticus* RS-1**	**Identity (%)**	−	68	−	−	−	−	−	−	−	−	−	−	72	−	−	−	−	−	−	−	−	−	−	−	−	−	−	−	−
	**Coverage (%)**	−	98	−	−	−	−	−	−	−	−	−	−	99	−	−	−	−	−	−	−	−	−	−	−	−	−	−	−	−
	***E*-value**	−	1e-95	−	−	−	−	−	−	−	−	−	−	0.0	−	−	−	−	−	−	−	−	−	−	−	−	−	−	−	−
	**Putative protein (Locus_tag)**	MamI-1 (NNJEOMEG_00106)	MamA (NNJEOMEG_00107)	MamI-2 (NNJEOMEG_00109)	MamQ (NNJEOMEG_00110)	MamB (NNJEOMEG_00112)	MamP-like (NNJEOMEG_00113)	MamE-Cter (NNJEOMEG_00114)	MamEO (NNJEOMEG_00115)	MamE-Nter (NNJEOMEG_00116)	MamI-3 (NNJEOMEG_00119)	MamL (NNJEOMEG_00120)	MamM (NNJEOMEG_00121)	MamK (NNJEOMEG_00129)	Mad1 (NNJEOMEG_00108)	Mad2 (NNJEOMEG_00111)	Mad4 (NNJEOMEG_00117)	Mad6 (NNJEOMEG_00118)	Mad7 (NNJEOMEG_00122)	Mad8 (NNJEOMEG_00123)	Mad9 (NNJEOMEG_00125)	Mad17 (NNJEOMEG_00126)	Mad30 (NNJEOMEG_00127)	Mad11 (NNJEOMEG_00128)	Mad10 (NNJEOMEG_00130)	Mad23 (NNJEOMEG_00134)	Mad25 (NNJEOMEG_00136)	Mad26 (NNJEOMEG_00137)	Mad28 (NNJEOMEG_00138)	Mad29 (NNJEOMEG_00141)

The above table shows amino acid sequence similarities including *E*-value and coverage with high sequence identities of known Mam and Mad proteins of other magnetotactic bacteria using blastx.

## Discussion

The present study showed that a sulfate-reducing magnetotactic bacterium, which we designated “strain FSS-1”, is considered to be a novel species of the genus *Desulfovibrio* affiliated with *Deltaproteobacteria*, and synthesizes magnetite nanoparticles in the cells. To our knowledge, cultured sulfate-reducing MTB belonging to the *Deltaproteobacteria* are present in 2 genera and 2 species; that is, *Desulfovibrio magneticus* strain RS-1^T^ and *Desulfamplus magnetovallimortis* strain BW-1^T^, which have already been validly accepted, whereas it was recently reported that other cultured sulfate-reducing MTB affiliated with the genus *Desulfovibrio*; strain FH-1 and strain ZBP-1, are, respectively, considered as strains of *D*. *magneticus* and *D*. *putealis* [31]. Note that the morphology of the bullet-shaped magnetite nanoparticles produced by WYHR-1 is quite different from that of the magnetite nanoparticles produced by *Deltaproteobacteria* and magnetotactic *Nitrospirae* as mentioned although WYHR-1 has not yet been cultured [20]. Thus, the strain FSS-1 is considered to be the second novel species of the genus *Desulfovibrio* that synthesizes magnetite nanoparticles. Although the magnetic collection and microscopic observation of MTB from natural environments are relatively easy, it is difficult to isolate and cultivate MTB using growth media since they are a fastidious member of prokaryotes and therefore some special culture conditions are required [58]. It is in particular difficult to isolate sulfate-reducing MTB since they synthesize very few magnetite nanoparticles and display only a weak magnetotactic response when they are grown in culture media [15, 20, 59, 60]. Hydrogen sulfide produced during sulfate reduction interferes with the formation of magnetosomes due to the extracellular precipitation of ferrous sulfide [5, 15]. *D*. *magneticus* also produces very few magnetite nanoparticles due to the formation of hydrogen sulfide in the cells during the growth [59]. In the present study, we found that despite sulfate reduction, almost all of the FSS-1 cells synthesized magnetite nanoparticles and responded to an external magnetic field although the final cell concentrations were rather low (Fig 3C). It is supposed that the strain FSS-1 successfully grew using iron ions for the synthesis of magnetite nanoparticles since the low cell concentrations prevented the production of an excess amount of precipitates of ferrous sulfide.

Bullet-shaped magnetite nanoparticles has been found in cultured and uncultured MTB affiliated only with the *Deltaproteobacteria* classes of *Proteobacteria* phylum, and with the *Nitrospirae* and “*Candidatus* Omnitrophica” phyla [23, 24]. Presently discovered strain FSS-1, which belongs to the *Deltaproteobacteria*, synthesized bullet-shaped magnetite nanoparticles in the cells (Fig 1B). The average number of bullet-shaped magnetite nanoparticles per cell in strain FSS-1 was 9.4, which is higher than that of *D*. *magneticus*; i.e., 6, when grown anaerobically (Fig 4B) [30]. The average length of bullet-shaped magnetite nanoparticles in strain FSS-1 was 53.9 nm, which is greater than that of *D*. *magneticus*; approximately 40 nm, when grown anaerobically (Fig 4C) [59]. Note that the growth conditions of FSS-1 were different from those of *D*. *magneticus* and that the number and size of bullet-shaped magnetite nanoparticles synthesized by *D*. *magneticus* were changed depending upon the growth conditions [31].

The easy, intermediate and hard directions of magnetization in cubic magnetite are, respectively, <111>, <110> and <100>. Thus, the most efficient arrangement for the magnetocrystalline anisotropy energy is the <111> direction along the elongation direction of the particle since the particle has the maximum magnetic moment per unit volume in the <111> direction [61]. It was shown that in the MTB belonging to the *Alphaproteobcteria* class, the magnetite nanoparticles in the cells was elongated in the <111> direction [24]. However, there are quite a few reports that the elongation of the bullet-shaped magnetite nanoparticles occurred in other directions rather than the <111> direction [16, 24, 27, 62–65]. It was revealed that in the case of *D*. *magneticus* and magnetotactic *Deltaproteobacteria* strain WYHR-1, the bullet-shaped magnetite nanoparticles in the cells were elongated along the <100> direction [8, 20, 59]. In the case of the presently discovered strain FSS-1 belonging to the genus *Desulfovibrio*, the elongation direction of the bullet-shaped magnetite nanoparticles in the cells also appears to be in parallel to the <100> direction (Fig 5E and 5F).

Comparative analysis of MGC showed that the MGC of strain FSS-1 differed from that of *Desulfovibrio magneticus* strain RS-1 (Fig 6). The absence of transposase genes or insertion elements indicates that the MGC of strain FSS-1 does not represent a genomic island such as that of *D*. *magneticus* strain RS-1 [57]. Due to the draft genome, however, we presently do not have enough information to determine whether the MGC of strain FSS-1 represents a genomic island or not. The G+C content of the 13 *mam* genes of strain FSS-1 was 64.2%, which showed a clear difference from that of *D*. *magneticus* strain RS-1; 62.8% [56]. Based on 16S rRNA gene sequence analysis, the sequence of the strain FSS-1 showed 96.6%, 91.6% and 91.4% similarities, respectively, to *Desulfovibrio sp*. strain ZBP-1, *Desulfovibrio sp*. strain FH-1 and *D*. *magneticus* strain RS-1^T^. Thus, these results support the conclusion that strain FSS-1 is a novel sulfate-reducing magnetotactic bacterium belonging to the genus *Desulfovibrio*. The *mad* genes found in the MGC, specific to magnetotactic *Deltaproteobacteria* and *Nitropirae*, are responsible for the formation of the magnetosomes composed of greigite and/or bullet-shaped magnetite nanoparticles [56]. Some of the functions of the *mad* genes have been elucidated, but most of the functions are still unclear [56, 66]. The MGC of our isolate may well play a key element in elucidating each function of the *mad* genes for the formation of the magnetosomes and provide an opportunity to address some important issue concerning the origin and evolution of magnetotaxis.

## Supporting information

S1 Fig Cultivation of strain FSS-1 under microaerobic and anaerobic conditions in a semi-solid medium. (A) Strain FSS-1 was cultivated under microaerobic conditions in a semi-solid medium containing 35 μM FeCl_2_•4H_2_O in the presence of 5.3 mM sodium sulfate. The semi-solid medium was composed of 0.5 mL/liter of modified Wolfe’s mineral elixir (all of the sulfate salts were replaced with chloride salts), 0.5 mg/liter of resazurin, 0.17 g/liter of NaNO_3_, 0.33 g/liter of succinic acid, and 0.082 g/liter of MgCl_2_•6H_2_O and the pH of the medium was adjusted to 7.0. The medium was solidified by 1.0 g/liter of Agar Noble (Becton, Dickinson and Company) instead of using agarose. After having autoclaved the medium, 0.5 ml/liter of a sterile anaerobic stock of vitamin solution, 5.6 ml/liter of a sterile anaerobic stock of 0.25 mM KHPO_4_ buffer (pH 7.0), 2.0 ml/liter of a sterile anaerobic stock of 5% NaHCO_3_, 3.5 ml/liter of a sterile anaerobic stock of 10 mM FeCl_2_•4H_2_O (in 0.02 N HCl), and 0.2 g/liter of freshly made neutralized and filter sterilized cysteine•HCl•H_2_O were added to the medium. Air was contained in the headspace of the tube. Strain FSS-1 was inoculated at the oxic-anoxic interface (OAI) of the semi-solid medium and cultivated at 28°C in dim light. No growth of FSS-1 was observed. (B) Strain FSS-1 was cultivated under microaerobic conditions in the semi-solid medium containing 35 μM FeCl_2_•4H_2_O in the absence of sodium sulfate. Air was contained in the headspace of the tube. Strain FSS-1 was inoculated at the OAI of the semi-solid medium and cultivated at 28°C in dim light. No growth of FSS-1 was observed. (C) Strain FSS-1 was cultivated under anaerobic conditions in the semi-solid medium containing 100 μM FeCl_2_•4H_2_O in the presence (left) or absence (right) of 5.3 mM sodium sulfate. The headspace vapor of the tube was replaced with 100% O_2_ free-N_2_ gas. Strain FSS-1 was inoculated into the semi-solid medium and cultivated at 28°C in dim light. FSS-1 grew only in the presence of sulfate (left).
(TIF)

S2 Fig STEM-EDS analysis of electron-dense precipitates from a culture of strain FSS-1. STEM-EDS spot analysis of central ((i), (iii) and (v)) and peripheral ((ii), (iv) and (vi)) areas of 3 different electron-dense precipitates ((A), (B) and (C)) indicated by asterisks is shown. Copper (Cu) signals are due to the TEM grid used, whereas the sodium (Na), magnesium (Mg), chlorine (Cl) and potassium (K) signals are from the culture medium.
(TIF)

S3 Fig STEM-EDS analysis of magnetic nanoparticles in a cell. (A) (i), (ii) STEM-EDS spot spectra at the center of a magnetic particle and a peripheral area indicated by asterisks in panel (A). Copper (Cu) signals are due to the TEM grid used.
(TIF)

S1 Table Final cell concentrations and the number of magnetic nanoparticles synthesized in strain FSS-1 grown using a single amino acid as an electron donor***. (DOCX)
