# Supplementary figures and images for: Isolation and cultivation of a novel sulfate-reducing magnetotactic bacterium belonging to the genus Desulfovibrio
Source: PLoS One. 2021 Mar 11;16(3):e0248313. doi: 10.1371/journal.pone.0248313 (PMC7951924; doi:10.1371/journal.pone.0248313)

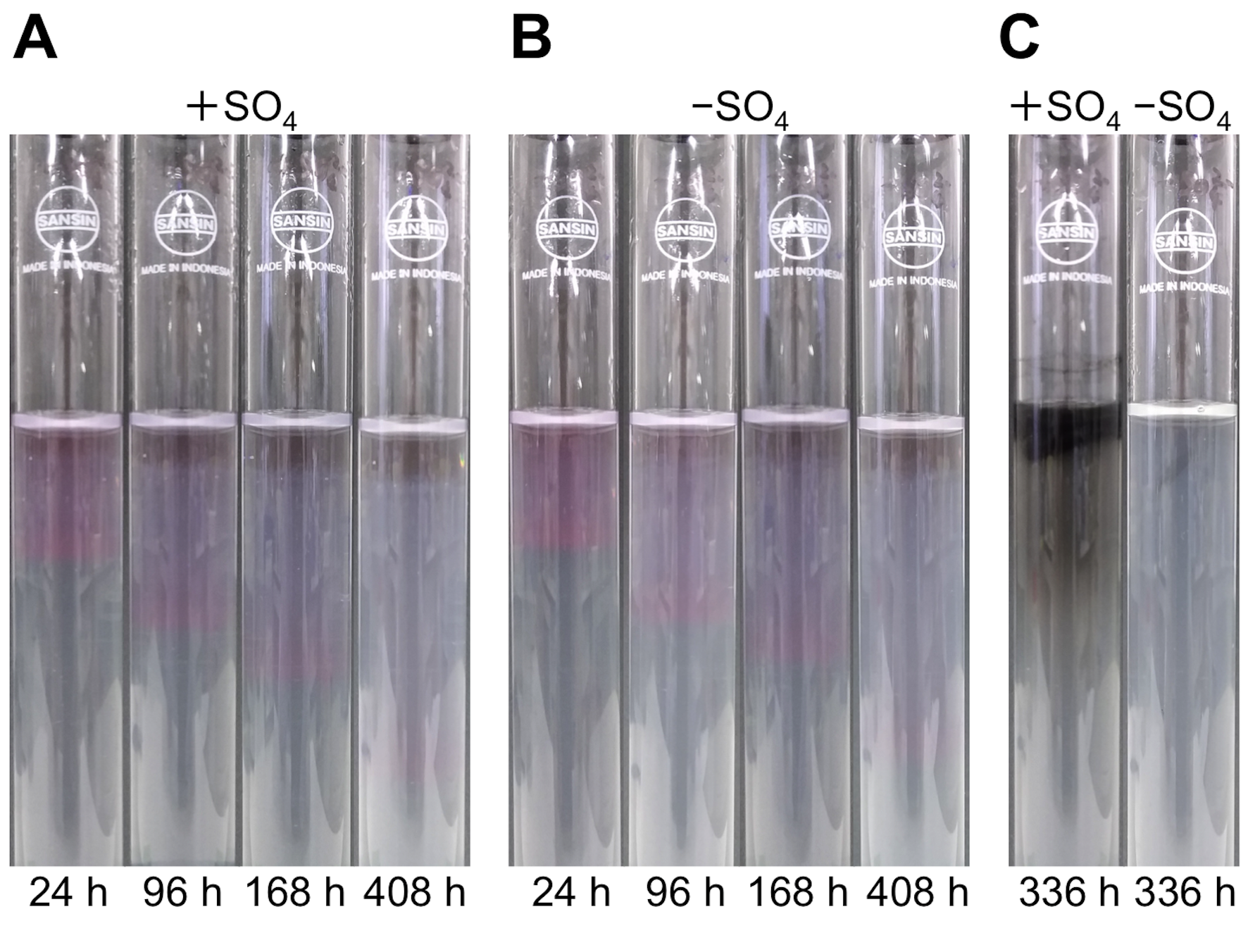

Supplement: S1 Fig — (A) Strain FSS-1 was cultivated under microaerobic conditions in a semi-solid medium containing 35 μM FeCl2•4H2O in the presence of 5.3 mM sodium sulfate. The semi-solid medium was composed of 0.5 mL/liter of modified Wolfe’s mineral elixir (all of the sulfate salts were replaced with chloride salts), 0.5 mg/liter of resazurin, 0.17 g/liter of NaNO3, 0.33 g/liter of succinic acid, and 0.082 g/liter of MgCl2•6H2O and the pH of the medium was adjusted to 7.0. The medium was solidified by 1.0 g/liter of Agar Noble (Becton, Dickinson and Company) instead of using agarose. After having autoclaved the medium, 0.5 ml/liter of a sterile anaerobic stock of vitamin solution, 5.6 ml/liter of a sterile anaerobic stock of 0.25 mM KHPO4 buffer (pH 7.0), 2.0 ml/liter of a sterile anaerobic stock of 5% NaHCO3, 3.5 ml/liter of a sterile anaerobic stock of 10 mM FeCl2•4H2O (in 0.02 N HCl), and 0.2 g/liter of freshly made neutralized and filter sterilized cysteine•HCl•H2O were added to the medium. Air was contained in the headspace of the tube. Strain FSS-1 was inoculated at the oxic-anoxic interface (OAI) of the semi-solid medium and cultivated at 28°C in dim light. No growth of FSS-1 was observed. (B) Strain FSS-1 was cultivated under microaerobic conditions in the semi-solid medium containing 35 μM FeCl2•4H2O in the absence of sodium sulfate. Air was contained in the headspace of the tube. Strain FSS-1 was inoculated at the OAI of the semi-solid medium and cultivated at 28°C in dim light. No growth of FSS-1 was observed. (C) Strain FSS-1 was cultivated under anaerobic conditions in the semi-solid medium containing 100 μM FeCl2•4H2O in the presence (left) or absence (right) of 5.3 mM sodium sulfate. The headspace vapor of the tube was replaced with 100% O2 free-N2 gas. Strain FSS-1 was inoculated into the semi-solid medium and cultivated at 28°C in dim light. FSS-1 grew only in the presence of sulfate (left). (TIF) [file pone.0248313.s001.tif]

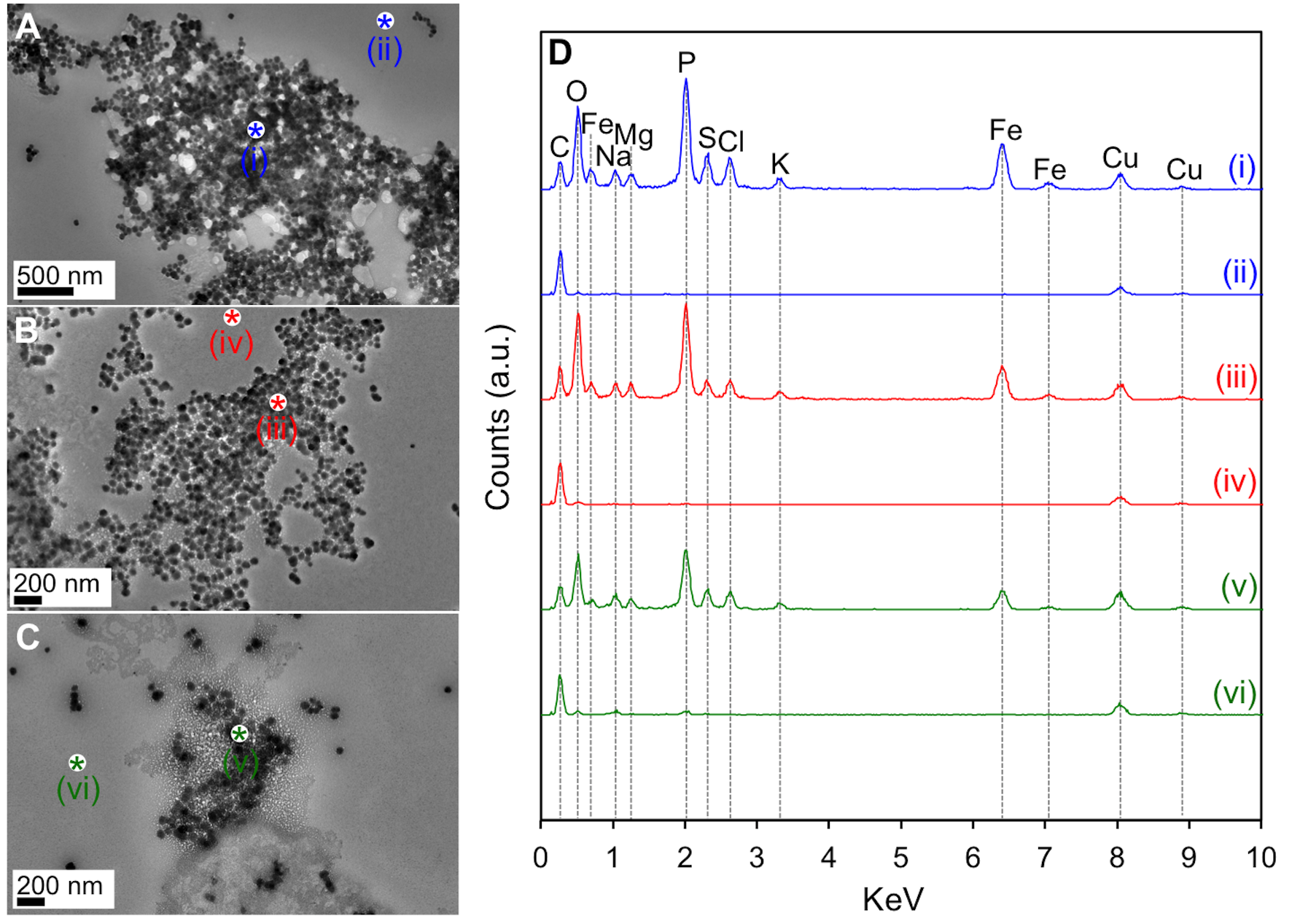

Supplement: S2 Fig — STEM-EDS spot analysis of central ((i), (iii) and (v)) and peripheral ((ii), (iv) and (vi)) areas of 3 different electron-dense precipitates ((A), (B) and (C)) indicated by asterisks is shown. Copper (Cu) signals are due to the TEM grid used, whereas the sodium (Na), magnesium (Mg), chlorine (Cl) and potassium (K) signals are from the culture medium. (TIF) [file pone.0248313.s002.tif]

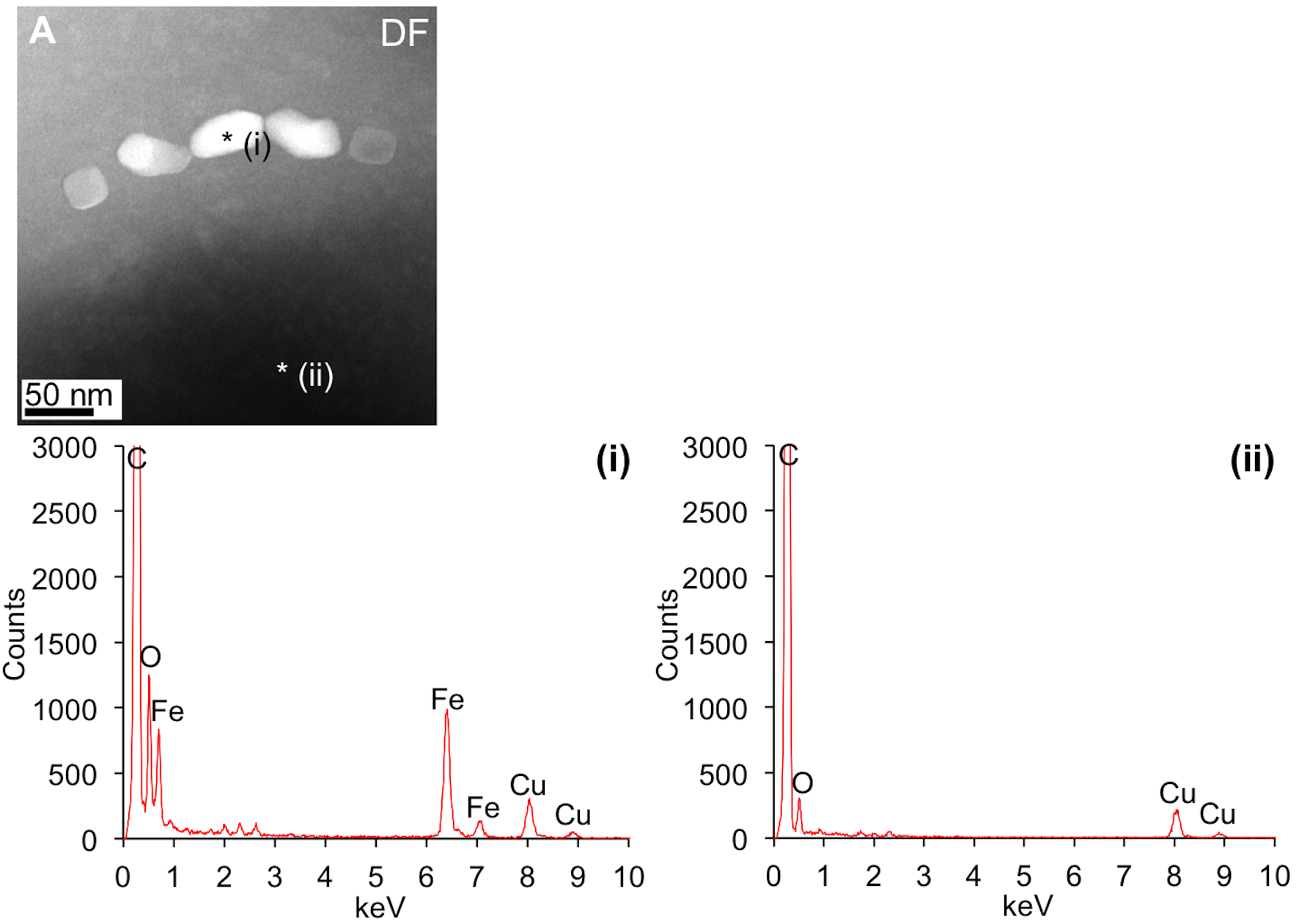

Supplement: S3 Fig — (A) (i), (ii) STEM-EDS spot spectra at the center of a magnetic particle and a peripheral area indicated by asterisks in panel (A). Copper (Cu) signals are due to the TEM grid used. (TIF) [file pone.0248313.s003.tif]
