# Supplementary material for: Isolation and cultivation of a novel sulfate-reducing magnetotactic bacterium belonging to the genus Desulfovibrio
Source: PLoS One. 2021 Mar 11;16(3):e0248313. doi: 10.1371/journal.pone.0248313 (PMC7951924; doi:10.1371/journal.pone.0248313)
Supplement: S1 Table — (DOCX) [file pone.0248313.s004.docx]

**S1 Table. Final cell concentrations and the number of magnetic nanoparticles synthesized in strain FSS-1 grown using a single amino acid as an electron donor^***^.**

|  | **Final cell concentration (cells/mL)** | | **Number of magnetic nanoparticles per cell^*^** | |
| --- | --- | --- | --- | --- |
| **Substrates** | **1 mM** | **5 mM** | **1 mM** | **5 mM** |
| Asparaginic acid | 3.4 × 10^5^ | 8.8 × 10^4^ | 8.7 (± 2.7) | 7.1 (± 3.3) |
| Glycine | 9.6 × 10^4^ | ––^**^ | 14.3 (± 9.5) | ––^**^ |
| Tryptophan | 1.4 × 10^5^ | 1.0 × 10^5^ | 13.7 (± 6.0) | 12.7 (± 3.2) |
| Valine | 1.2 × 10^5^ | 8.4 × 10^4^ | 11.4 (± 4.0) | 11.6 (± 3.2) |

^*^ The number of magnetic nanoparticles in each cell was counted targeting at 50 individual cells.

^**^No cell growth.

^***^In order to understand the growth of strain FSS-1 in more detail, the effect of individual amino acids on the growth of strain FSS-1 was investigated. 1 and 5 mM of an individual amino acid such as alanine, arginine, asparagine, asparaginic acid, isoleucine, glutamine, glutamic acid, glycine, serine, tyrosine, tryptophan, threonine, valine, histidine, phenylalanine, proline, methionine, lycine and leucine, was added as an electron donor to a casamino acids-free and yeast extract-free MD medium. Note that cysteine was contained as a reducing agent in the MD medium and we confirmed that the sulfate reduction was not induced by cysteine. The cultivation procedure was as follows; (1) The initiation of cultures was carried out in the MD medium containing 0.1 g/liter of sodium formate in place of casamino acids for 14 days. The final cell concentration reached 2.3 × 10^6^ cells/mL. (2) The growth cultures were transferred three times into fresh media containing the same amino acid to remove formate and yeast extract remaining in the cultures. Note that the inoculum, the concentration of which in the MD medium was 0.5 % (vol/vol), was grown each time the growth cultures were transferred into the fresh media. (3) The growth of strain FSS-1 in the presence of an individual amino acid was analyzed after 21 day incubation, comparing it with the growth in the absence of an amino acid.
